# Evaluation of Acidic Ionic Liquids as Catalysts for Furfural Production from *Eucalyptus nitens* Wood

**DOI:** 10.3390/molecules27134258

**Published:** 2022-07-01

**Authors:** Lucía Penín, Mar López, Valentín Santos, Juan Carlos Parajó

**Affiliations:** CINBIO, Department of Chemical Engineering, Faculty of Science, University of Vigo (Ourense Campus), Polytechnical Building, As Lagoas, 32004 Ourense, Spain; lpenin@uvigo.es (L.P.); marlopezr@uvigo.es (M.L.); vsantos@uvigo.es (V.S.)

**Keywords:** *Eucalyptus nitens*, acidic ionic liquid, biphasic system, methyl isobutyl ketone, furfural

## Abstract

*Eucalyptus nitens* wood samples were subjected to hydrothermal processing to obtain soluble saccharides from the hemicellulosic fraction. The hemicellulose-derived saccharides were employed as substrates for furfural production in biphasic media made up of water, methyl isobutyl ketone, and one acidic ionic liquid (1-butyl-3-methylimidazolium hydrogen sulfate or 1-(3-sulfopropyl)-3-methylimidazolium hydrogen sulfate). The reactions were carried out in a microwave-heated reactor to assess the effects of the most influential variables. Under selected operational conditions, the molar conversions of the precursors into furfural were within the range of 77–86%. The catalysts conserved their activity after reutilization in five consecutive reaction cycles.

## 1. Introduction

Sustainability and environmental concerns derived from the intensive use of fossil resources foster the use of renewable resources as feedstocks for the industry. Among these problems, climate change is one of the main ones to combat. Other problems linked to the intensive use of fossil resources are their limited availability, supply insecurity, and the volatility of prices. In this context, the European Commission has adopted the European Green Deal [1], a strategy to address the current climate and environmental challenges.

Vegetal biomass, and particularly lignocellulosic materials (LCMs), are renewable and potential feedstock for the industry that could help to meet the objectives of a carbon-neutral economy by 2050, as proposed by the European Commission. These materials can be treated following the biorefinery approach. According to the International Energy Agency [2], a biorefinery is a facility that, operating in an analogous way to an oil refinery, sustainably and synergistically processes all the components of biomass into a wide spectrum of products (including chemicals and materials), marketable food and feed ingredients, and energy (fuel, power, and heat).

Interestingly, integrated biorefineries (capable of obtaining benefits from the diverse LCM components) are compatible with the circular economy principles [3] and have been claimed to facilitate the transition to a circular economy on the basis of suitable waste management [4].

Among the multiple types of LCM available for industrial processing, hardwoods show important advantages, such as limited lignin percentages, satisfactory polysaccharide content, and hemicelluloses typically dominated by heteroxylan. *Eucalyptus*, the most widely planted type of hardwood, has been proposed as a feedstock for industrial processes based on sustainable conversion technologies [5], favored by their ability to be produced at a relatively low cost [6]. In this field, *Eucalyptus nitens* is attracting interest owing to its specific features, including comparatively good resistance to plagues [7,8,9] and low temperatures [7].

The fact that heteroxylan in *E. nitens* is the dominant hemicellulose polymer facilitates its separation from cellulose and lignin via aqueous processing with hot, compressed water (autohydrolysis or hydrothermal processing). Autohydrolysis is a green fractionation technology that stands out for its ability to convert hemicelluloses into soluble saccharides (low-molecular-weight polysaccharides, xylooligosaccharides, and xylose) at high yields while keeping cellulose and lignin in solid phase [10,11,12,13,14]. Multistage processes based on autohydrolysis achieve added value from the raw material through the fractionation of the constituent polymers and further conversion of the resulting compounds into chemicals, fuels, and/or materials. In this scope, the hemicellulose-derived saccharides from hardwood autohydrolysis are suitable substrates for furfural manufacture. The situation is more complex when softwoods (whose hemicellulose fraction contains both heteroxylan and glucomannan) are employed as feedstock for autohydrolysis, the soluble products generated include pentoses, saccharides made up of anhydropentoses, hexoses, and saccharides made up of anhydrohexoses. In this last case, sugar dehydration leads to furfural, as well as hydroxymethylfurfural, levulinic acid, and formic acid [13,14].

The major reactions involved in furfural manufacture based on the autohydrolysis of hardwood are as follows [10,12,13,14]:

(a) Partial hydrolysis of pentosans (xylan or arabinan, with formula (C_5_H_8_O_4_)_n_) via autohydrolysis, involving the uptake of 1 mol water/mol anhydrosugar, to yield soluble low-molecular-weight polysaccharides or oligosaccharides made up of anhydropentoses (with formula (C_5_H_8_O_4_)_k_, where n and k are the polymerization degrees of pentosans and soluble higher saccharides, respectively;
(b) Hydrolysis of the above low-molecular-weight polysaccharides or oligosaccharides made up of anhydropentoses into pentoses (xylose or arabinose, with formula C_5_H_10_O_5_, consuming 1 mol water/mol anhydrosugar);
(c) Dehydration of the above pentoses (xylose or arabinose) into furfural (C_5_H_4_O_2_), releasing 3 mol water/mol pentose.

Additionally, it can be noted that the solids from autohydrolysis (mainly made up of cellulose and lignin) are susceptible to further fractionation (for example, by pulping reactions), enabling a complete utilization of the raw material. This approach has been successfully assessed in literature for the kraft pulping of autohydrolyzed *Eucalyptus* wood [15,16].

Furfural (OC_4_H_3_CHO), a platform chemical with a bright future, has been mentioned as one of the top 30 value-added chemicals derived from biomass in a report supported by the US Department of Energy [17], further updated by Bozell and Petersen [18]. The interest in furfural relies on both its important current applications [19,20,21] and its role as a precursor of new families of bio-based, sustainable chemicals and fuels [22]. In particular, furfural is the sole precursor for all compounds containing a furyl, furfuryl, furoyl, or furfurylidene radical, such as furfuryl alcohol, furan, tetrahydrofurfuryl alcohol, furfurylamine, tetrahydrofurfurylamine, 2-methylfuran, 2-methyltetrahydrofuran, and furoic acid [23].

As indicated above, the production of furfural in acidic media from pentoses, pentosans, or higher soluble saccharides derived from pentosans involves the dehydration of pentoses or the hydrolysis–dehydration of oligomers or polymers containing anhydropentoses. However, a number of undesired, side reactions also occur, leading to decreased furfural yields [24,25]. A number of strategies have been proposed to improve the furfural yields, including the utilization of new catalysts of enhanced selectivity, improved reaction technologies, and continuous separation of the target product from the reaction phase.

Acidic ionic liquids (here denoted AIL) have been proposed as environmentally friendly catalysts for LCM biorefineries, based on their physicochemical properties (particularly, low volatility and good stability), which allow fewer emissions and an easier recovery [26,27]. Most studies on LCM fractionation with ionic liquids, including AIL, have been carried out with compounds bearing the imidazolium ion. In LCM biorefineries, imidazolium-type AIL can be employed as solvents, as acidic catalysts, or playing both roles simultaneously, and may increase conversion and selectivity values [28].

In recent years, microwave reactors have attracted attention for LCM processing owing to their advantages over conventional reactors, such as fast heating profiles (and, therefore, faster reaction rates), higher energy efficiency, and the possibility of improving the results, increasing the product yields and/or decreasing the generation of undesired byproducts [29,30].

In the manufacture of furfural from LCMs or LCM-derived saccharides using aqueous treatments in the presence of an acidic catalyst, the extent of undesired side reactions can be limited by transferring the target product to an immiscible organic solvent. The organic solvent should present favorable partition coefficients and low water solubility. These conditions are met by methyl isobutyl ketone (MIBK), an environmentally friendly solvent included in the CHEM21 guide [31], which has been employed in the literature for furfural production in media catalyzed with ionic liquids [32,33].

This study deals with the production of furfural from hemicellulose-derived saccharides obtained via the hydrothermal treatment of *E. nitens* wood. The operation was carried out in biphasic media containing the liquid phase from hydrothermal processing, MIBK, and an AIL acting as a catalyst. The catalyst was one of the following AIL: 1-butyl-3-methylimidazolium hydrogen sulfate, denoted [C4mim]HSO_4_; or 1-(3-sulfopropyl)-3-methylimidazolium hydrogen sulfate, denoted [C3SO_3_Hmim]HSO_4_. Reactions were performed in a stirred, microwave-heated reactor, and the effects of the selected variables (organic-to-aqueous phase-mass ratio, denoted OAR; catalyst concentration, denoted CC; and isothermal reaction time, denoted t) were assessed for optimal furfural production.

## 2. Results and Discussion

### 2.1. Wood Processing

The hydrothermal processing of *E. nitens* wood was performed as per Penín et al. [34]. In this study, the optimal conditions for *E. nitens* autohydrolysis (carried out in the same reactor and with the same hydromodule employed here) were optimized in terms of the severity parameter S, defined by Overend and Chornet [35] as

(1) $$\mathrm{Severity}=\log\left[\int_{T_{0}}^{T_{\max}}exp\left(\frac{T\left(t\right)-T_{\mathrm{ref}}}{T_{\omega }}\right)\cdot \mathrm{dt}\right]$$

where T_0_ is the initial temperature; T_max_ is the maximum temperature; T(t) represents the temperature profile; T_ref_ is the reference temperature (100 °C); and T_ω_ is a parameter measuring the activation energy, which was fixed in the value (14.75 °C), as proposed by Overend and Chornet. The best conditions were found to correspond to a severity rate of 3.62 (corresponding to a maximum temperature of 195 °C).

The liquid phase from autohydrolysis performed under these conditions presented the following composition (in g/L): xylosyl groups in oligosaccharides, 15.1; xylose, 4.08; arabinose, 0.575; furfural, 0.248; acetyl groups in oligosaccharides, 3.95; acetic acid, 0.934; glucosyl groups in oligosaccharides, 1.19; glucose, 0.443; and 5-hydroxymethylfurfural, 0.034. Besides xylan-derived products (xylooligosaccharides, xylose resulting from their hydrolysis, and furfural coming from xylose dehydration), the medium also contained low concentrations of arabinose (produced from arabinan, a hemicellulose component), acetyl substituents in xylooligosaccharides, and acetic acid (produced by acetyl group hydrolysis). The rest of the components (glucosyl groups, glucose, and 5-hydroxymethylfurfural) were produced at low conversions by hydrolysis or hydrolysis–dehydration of glucose-containing polymers present in the native wood. It can be noted that some furfural can also be produced from the small amounts of arabinose detected since this sugar also undergoes dehydration in aqueous, acidic media [13,14].

The aqueous phase from *E. nitens* autohydrolysis was employed for furfural manufacture using an AIL as a catalyst. In this study, the experimental results were obtained using biphasic media (water/MIBK) containing one AIL ([C4mim]HSO_4_ or [C3SO_3_Hmim]HSO_4_) as a catalyst.

### 2.2. Experiments Using [C4mim]HSO_4_ as a Catalyst

Based on preliminary experiments (data not shown), the effects of the relative amount of organic solvent (OAR in the range of 1–4 g/g) were assessed by performing treatments at 180 °C for 30 min in media containing 1.16 mol [C4mim]HSO_4_/L aqueous phase. 

Table 1 shows that furfural was concentrated in the organic phase, owing to the favorable partition coefficient. For the sake of simplicity, the results in Table 1 are expressed in terms of conversions of potential substrates into furfural (FC, defined as g furfural/100 g of furfural resulting from the stoichiometric conversion of the precursors). The data are reported separately for the aqueous and organic phases, and the discussion is made in terms of the total FC. No significant amounts of formic acid were found in the media, confirming that the decomposition of furfural into formic acid was negligible under the conditions tested. 

The experimental data showed a slight FC increase when OAR increased from 1 to 2, almost no variation in FC when OAR increased from 2 to 3, and a small FC decrease when OAR increased from 3 to 4. Based on these results, OAR = 2 was considered optimal, owing to the good balance between furfural conversion, limitation of solvent usage, and increased volumetric concentration of the target product. Owing to this, the rest of the experiments were performed at OAR = 2 g/g.

**Figure 1:**
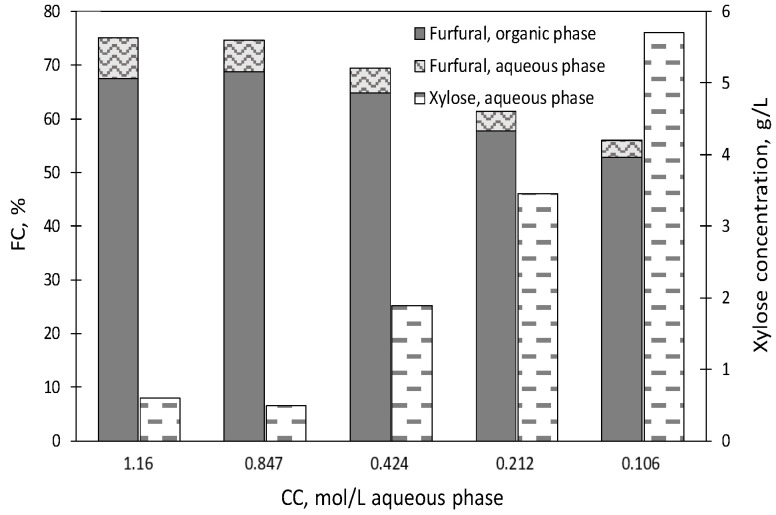
Molar conversions of potential substrates into furfural (FC) and xylose concentrations obtained at 180 °C using [C4mim]HSO_4_ as a catalyst.

**Figure 2:**
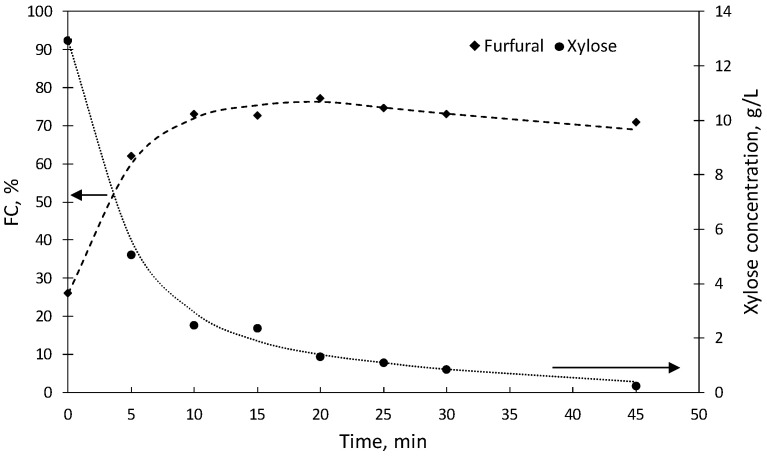
Molar conversions of potential substrates into furfural (FC) and xylose concentrations, obtained at 180 °C in experiments using [C4mim]HSO_4_ as a catalyst. Results were obtained at diverse reaction times in media containing 0.847 mol [C4mim]HSO_4_/L aqueous phase.

To assess the effects of the catalyst concentration, five additional experiments were performed at 180 °C for 30 min with OAR = 2, in media containing 0.106–1.16 mol [C4mim]HSO_4_/L (Figure 1 and Figure 2). The maximum FC (75.0 ± 0.71%) was reached in the assay performed at the highest catalyst concentration assayed (1.16 mol [C4mim]HSO_4_/L aqueous phase).

When the catalyst loading decreased to 0.847 mol [C4mim]HSO_4_/L aqueous phase, the concentrations of furfural in the aqueous and organic phases were 0.612 ± 0.015 and 3.48 ± 0.004 g/L, respectively (corresponding to molar conversions of 5.7 ± 0.14 and 68.8 ± 0.08%, respectively).

Lower catalyst loadings resulted in decreased furfural concentrations, as can be observed in Figure 1. Operating with 0.424 mol [C4mim]HSO_4_/L, FC reached 69.5 ± 0.46% (with furfural concentrations of 0.528 ± 0.012 and 3.28 ± 0.018 g/L in the aqueous and organic phases, respectively). The experiments performed with CC of 0.212 or 0.106 mol [C4mim]HSO_4_/L aqueous phase led to overall furfural molar conversions of 61.3 ± 0.07 and 55.9 ± 0.65%, respectively. Since no significant conversions were found when using catalyst concentrations of 1.16 or 0.847 mol/L aqueous phase (corresponding to FC values of 75.0 ± 0.71% and 74.5 ± 0.22%, respectively), further experiments were carried out while keeping CC constant at 0.847 mol/L aqueous phase.

As a result of biphasic treatments, most xylooligomers were converted into xylose, which appeared concentrated in the aqueous phase (Figure 1). Concerning the experimental error, the range observed for the absolute value of the deviations (|δ|) and the corresponding average values (|δ_a_|) were as follows: %FC in organic phase, |δ|range 0.02–0.71, |δ_a_|= 0.35; %FC in aqueous phase, |δ|range 0.00–0.14, |δ_a_|= 0.07; xylose concentration in g/L, |δ|range 0.01–0.51, |δ_a_|= 0.19.

Based on the above information, a new set of 8 assays were performed at 180 °C for reaction times up to 45 min in media formulated with 0.847 mol [C4mim]HSO_4_/L aqueous phase. Figure 2 shows the experimental results obtained in terms of FC, including the contributions of both aqueous and organic phases. Operating up to reach 180 °C with no isothermal stage (reaction time, 0 min), furfural was obtained at 26.1 ± 1.29% molar yield. Longer reaction times improved the furfural production: after 15 min, FC achieved 72.8 ± 0.34% and reached a maximum (77.4 ± 0.13%) after 20 min. Harsher conditions (reaction time, 25 min) resulted in decreased FC (74.8 ± 0.68%). The losses of furfural became more important at longer reaction times: for example, after 30 and 45 min, FC dropped to 73.1 ± 0.70 and 70.9 ± 0.96%, respectively. The deviations determined for the data in Figure 2 were as follows: %FC, |δ|range 0.13–1.29, |δ_a_|= 0.73; xylose concentration in g/L, |δ|range 0.00–0.38, |δ_a_|= 0.20.

In related studies dealing with the manufacture of furfural from commercial xylose in biphasic media, Peleteiro et al. [36] reported FC around 71% in water/toluene or MIBK/[C4mim]HSO_4_ media, whereas Wang et al. [37] achieved FC = 79.76% in media made up of water/γ-valerolactone/1-butyl-3-methylimidazolium chloroaluminate. Recently, Xia et al. [38] employed a biphasic medium made up of [C4mim]HSO_4_ (which served as the reaction phase and catalyst) and 1,4-dioxane for furfural manufacture from xylose or bamboo hemicelluloses under microwave irradiation. The addition of a small amount of water improved the furfural molar conversions up to 63.87% or 85.69% starting from xylose or bamboo hemicelluloses, respectively.

### 2.3. Experiments Using [C3SO_3_Hmim]HSO_4_ as a Catalyst

**Figure 3:**
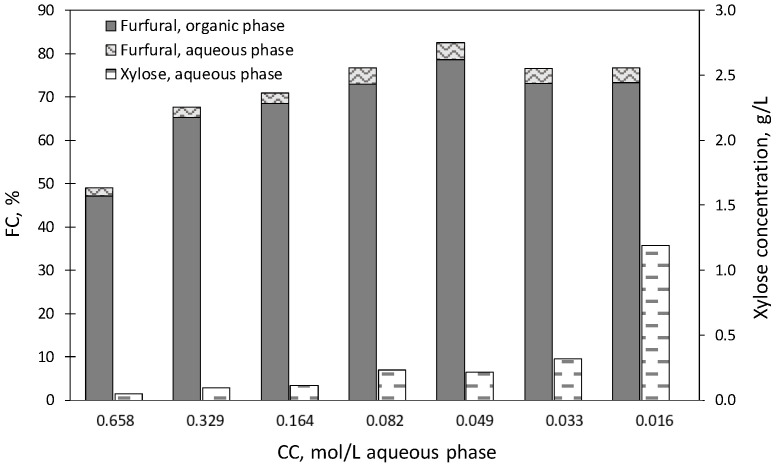
Molar conversions of precursors into furfural, and xylose concentrations in experiments performed at 180 °C using [C3SO_3_Hmim]HSO_4_ as a catalyst. Data were obtained for diverse catalyst concentrations in experiments lasting 20 min.

In order to assess the possible benefits derived from using a catalyst of higher acidity, a new set of experiments were performed using [C3SO_3_Hmim]HSO_4_ instead [C4mim]HSO_4_. For the sake of simplicity, the first experiment of the set was performed under the conditions (180 °C, 20 min, OAR = 2) identified as optimal in the previous section for operation with [C4mim]HSO_4_, and the effects of the catalyst charge were assessed. The experimental results are shown in Figure 3. Operating with CC = 0.658 mol/L liquid phase, furfural reached poor concentrations in the aqueous and organic phases (0.207 ± 0.002 and 2.38 ± 0.072 g/L, respectively; corresponding to FC = 1.9 ± 0.02 and 47.1 ± 1.42%), confirming that the increased catalytic activity of [C3SO_3_Hmim]HSO_4_ boosted the furfural-consuming reactions and suggesting that milder operational conditions would result in improved furfural production. Following this idea, a new experiment was performed cutting the catalyst concentration by half (CC = 0.329 mol/L liquid phase). Although the furfural concentrations in both phases increased by factors of 1.31–1.38, the resulting FC (67.6 ± 1.56%) was still below the target range, suggesting that lower catalyst charges could improve the results. To confirm this idea, a new set of experiments were performed with lower catalyst concentrations (CC = 0.164, 0.082, 0.049, 0.033 or 0.016 mol [C3SO_3_Hmim]HSO_4_/L aqueous phase), keeping the rest of operational variables constant. When CC decreased from 0.164 to 0.049 mol [C3SO_3_Hmim]HSO_4_/L, FC increased from 70.9 ± 0.30 up to 82.5 ± 0.91%, whereas lower CC (0.033 or 0.016 mol [C3SO_3_Hmim]HSO_4_/L) led to decreased yields (below 77%). The deviations observed for the data in Figure 3 were as follows: %FC in organic phase, |δ|range 0.24–1.54, |δ_a_|= 0.88; %FC in aqueous phase, |δ|range 0.00–0.19, |δ_a_|= 0.07; xylose concentration in g/L, |δ|range 0.00–0.04, |δ_a_|= 0.01.

Based on these results, CC = 0.049 mol [C3SO_3_Hmim]HSO_4_/L was considered optimal and, therefore, was employed in further experiments.

**Figure 4:**
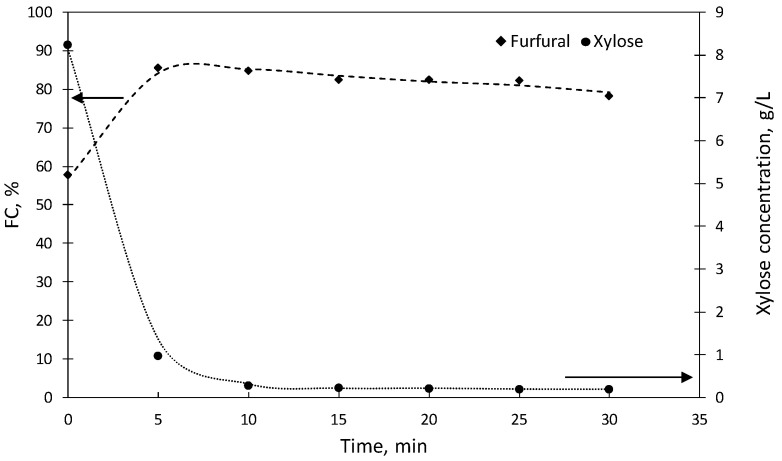
Molar conversions of precursors into furfural, and xylose concentrations in experiments performed at 180 °C using [C3SO_3_Hmim]HSO_4_ as a catalyst. Data were obtained at diverse reaction times in media containing 0.049 mol catalyst/L aqueous phase.

In order to assess the effects of the reaction time, experiments lasting up to 30 min were performed in media containing the optimal catalyst concentration, keeping the rest of the operational variables unchanged. The results (Figure 4) led to a poor FC (57.8 ± 2.37%) when the operation was performed up to reach 180 °C, without an isothermal stage (reaction time = 0). The maximum FC (85.6 ± 0.32%) was achieved after 5 min, whereas longer reaction times (up to 30 min) resulted in decreased furfural concentrations (with FC = 78.3 ± 0.52% after 30 min). Interestingly, the [C3SO_3_Hmim]HSO_4_ concentration leading to the highest FC was lower than in the previous case, as a consequence of its higher activity. The deviations observed for the data in Figure 4 were as follows: %FC, |δ|range 0.32–2.37, |δ_a_|= 0.88; xylose concentration in g/L, |δ|range 0.01–0.54, |δ_a_|= 0.09.

[C3SO_3_Hmim]HSO_4_ has been employed in the literature as a catalyst for furfural manufacture from commercial substrates. Matsagar et al. [39] employed a water–toluene system (1:5 *v/v*) containing 3% or 6% xylose at 170 °C, to obtain furfural at FC = 81 and 73%, respectively, after 4 h. Lin et al. [40] assessed the effects of different SO_3_H-functionalized ionic liquids (including [C3SO_3_Hmim]HSO_4_) as catalysts for furfural manufacture from xylose in water–GVL. The best result (FC = 78.12%) was obtained using 1-propylsulfonic-3-methylimidazolium chloride at 140 °C for 180 min. Recently, López et al. [41] obtained furfural from hemicellulose hydrolyzates, which were processed with [C3SO_3_Hmim]HSO_4_ and MIBK to yield furfural at near 78% molar conversion.

### 2.4. Catalyst Recycling

The possibility of reusing the catalyst in consecutive reaction cycles was assessed from reaction media obtained under the conditions considered optimal (180 °C, 20 min, 0.847 mol [C4mim]HSO_4_/L or 0.049 mol [C3SO_3_Hmim]HSO_4_/L aqueous phase, 180 °C, 5 min). The organic and aqueous phases obtained in the experiment were separated via decantation, and the aqueous phase (containing the catalyst together with the target product and byproducts) was filtered. The filtrate was extracted with ethyl acetate, and subjected to vacuum evaporation to recover the catalyst as the non-volatile fraction. The recovered catalyst was employed in further experiments (after supplementation with fresh AIL when necessary to compensate for losses).

**Figure 5:**
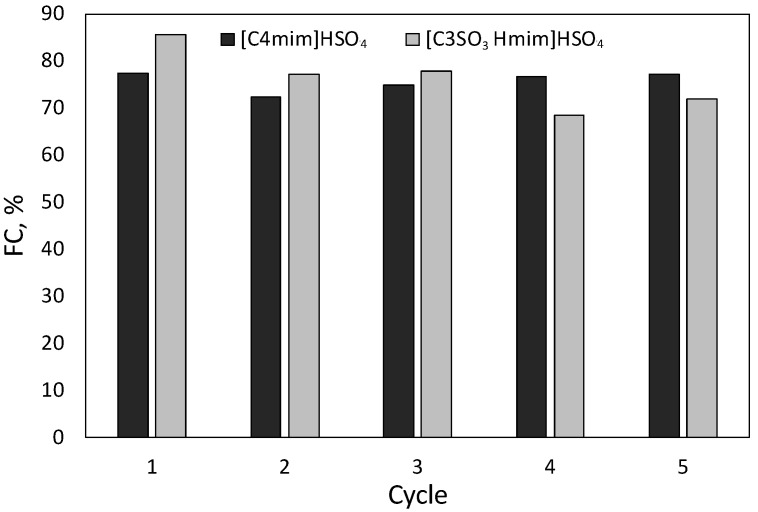
Molar conversion into furfural determined for 5 consecutive reaction cycles using [C4mim]HSO_4_ or [C3SO_3_Hmim]HSO_4_.

Figure 5 shows the experimental results obtained for consecutive experiments using recovered catalysts. The experiments using recovered [C4mim]HSO_4_ led to FC in the range 72.4–77.4% (average value, 75.7%), confirming that the catalyst kept most of its activity after five runs. The assays performed with recovered [C3SO_3_Hmim]HSO_4_ resulted in FC in the range 68.5–85.6% (average value, 76.2%). The deviations observed for the data in Figure 4 were as follows: %FC for experiments with [C4mim]HSO_4_, |δ|range 0.17–3.32, |δ_a_|= 1.45; %FC for experiments with [C3SO_3_Hmim]HSO_4_, |δ|range 0.19–0.55, |δ_a_|= 0.34; xylose concentration in g/L, |δ|range 0.01–0.54, |δ_a_|= 0.09. The increased experimental error observed in these experiments was ascribed to the utilization of very small amounts of catalyst.

In conclusion, high furfural conversions were obtained from the hemicellulose fraction of *E. nitens* wood operating in biphasic media catalyzed using [C4mim]HSO_4_ or [C3SO_3_Hmim]HSO_4_. The conversion of potential substrates into furfural was in the range of 77–86%. These results showed that the comparatively high acidity of [C3SO_3_Hmim]HSO_4_ enabled operation under mild conditions (defined by decreased catalyst concentrations and isothermal reaction times). Both AIL considered in this study kept most of their catalytic activity after reutilization in five consecutive reaction cycles.

## 3. Materials and Methods

### 3.1. Materials and Reaction in Biphasic Media

**Figure 6:**
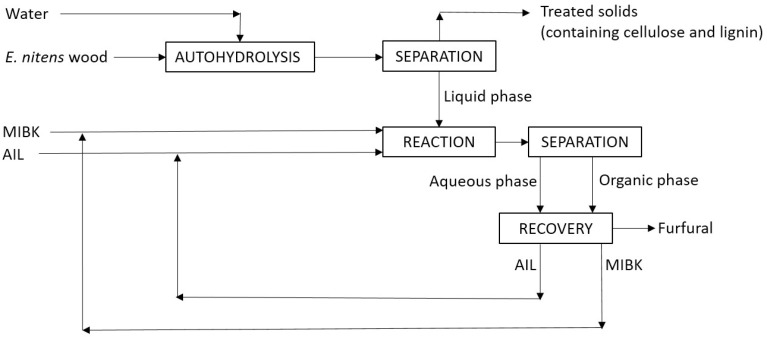
Scheme of wood processing for furfural production.

*E*. *nitens* wood samples were provided by ENCE (Pontevedra, Spain). Hydrothermal treatments were performed under the conditions reported as optimal [20]. The liquid phases from hydrothermal treatments were mixed with MIBK and one of the catalysts ([C4mim]HSO_4_ or [C3SO_3_Hmim]HSO_4_), and processed in a stirred MARS 6 microwave reactor under the desired reactions conditions. The reactions and the analyses were performed in duplicate. The experimental tasks are summarized in Figure 6.

### 3.2. Analytical Methods

The liquid phases from hydrothermal treatments (as collected, or after quantitative saccharification to convert the higher saccharides into monosaccharides), as well as the aqueous and organic phases from furfural production runs, were analyzed via HPLC operating as per Peleteiro et al. [19].

### 3.3. AIL Reutilization

The aqueous phase from the first reaction medium obtained in the set of experiments was filtered through a 0.2 µm membrane, extracted with ethyl acetate, and subjected to evaporation in a vacuum oven at 50 °C for 48 h. The AIL recovered as the non-volatile fraction was supplemented with fresh catalyst when necessary to compensate for losses and employed in the next run.

## Figures and Tables

**Table 1:** Conversions of the potential substrates into furfural (FC) in the aqueous and organic phases obtained at 180 °C, operating for 30 min with [C4mim]HSO_4_ charge of 1.16 mol/L aqueous phase, using OAR in the range 1–4 g/g.

Experiment	OAR (g/g)	FC (%)		
		Aqueous Phase	Organic Phase	Total
1	1	12.4 ± 0.11	60.1 ± 0.50	72.5 ± 0.39
2	2	7.5 ± 0.004	67.5 ± 0.71	75.0 ± 0.71
3	3	4.9 ± 0.06	70.4 ± 0.57	75.3 ± 0.51
4	4	3.4 ± 0.01	71.2 ± 0.26	74.6 ± 0.27

## Data Availability

Not applicable.

## Sample Availability

Samples of the compounds are not available from the authors.

## References

[1] European Commission (2019). The European Green Deal. Communication from the Commission to the European Parliament, the European Council, the Council, the European Economic and Social Committee and the Committee of the Regions. Brussels. https://eur-lex.europa.eu/resource.html?uri=cellar:b828d165-1c22-11ea-8c1f-01aa75ed71a1.0002.02/DOC_1&format=PDF.

[2] International Energy Agency (2014). IEA Bioenergy Task 42 Biorefining. Sustainable and Synergetic Processing of Biomass into Marketable Food & Feed Ingredients, Chemicals, Materials and Energy (Fuels, Power, Heat). https://www.ieabioenergy.com/wp-content/uploads/2014/09/IEA-Bioenergy-Task42-Biorefining-Brochure-SEP2014_LR.pdf.

[3] Liguori R., Faraco V. (2016). Biological processes for advancing lignocellulosic waste biorefinery by advocating circular economy. Bioresour. Technol..

[4] Velvizhi G., Balakumar K., Shetti N.P., Ahmad E., Pant K.K., Aminabhavi T.M. (2022). Integrated biorefinery processes for conversion of lignocellulosic biomass to value added materials: Paving a path towards circular economy. Bioresour. Technol..

[5] Cherubini F., Strømman A.H. (2011). Life cycle assessment of bioenergy systems: State of the art and future challenges. Bioresour. Technol..

[6] Magaton A.D.A.S., Colodette J.L., De Gouvêa A.F.G., Gomide J.L., Muguet M.C.D.S., Pedrazzi C. (2009). *Eucalyptus* wood quality and its impact on kraft pulp production and use. TAPPI J..

[7] Pérez S., Renedo C.J., Ortiz A., Mañana M., Silió D. (2006). Energy evaluation of the *Eucalyptus globulus* and the *Eucalyptus nitens* in the north of Spain (Cantabria). Thermochim. Acta.

[8] Valente C., Gonçalves C.I., Monteiro F., Gaspar J., Silva M., Sottomayor M., Paiva M.R., Branco M. (2018). Economic outcome of classical biological control: A case study on the *Eucalyptus* snout bettle, *Gonipterus platensis*, and the parasitoid *Anaphes nitens*. Ecol. Econ..

[9] Pérez-Cruzado C., Muñoz-Sáez F., Basurco F., Riesco G., Rodríguez-Soalleiro R. (2011). Combining empirical models and the process-based model 3-PG to predict *Eucalyptus nitens* plantations growth in Spain. Forest Ecol. Manag..

[10] Gullón B., Dávila I., García-Torreiro M., Yáñez R., Labidi J., Gullón P., Ruiz H.A., Thomsen H.T., Trajano H.L. (2017). Production and emerging applications of bioactive oligosaccharides from biomass hemicelluloses by hydrothermal processing. Hydrothermal Processing in Biorefineries: Production of Bioethanol and High Added-Value Compounds of Second and Third Generation Biomass.

[11] Michelin M., Romaní A., Salgado J.M., Domingues L., Teixeira J.A., Ruiz H.A., Thomsen H.T., Trajano H.L. (2017). Production of hemicellulases, xylitol, and furan from hemicellulosic hydrolysates using hydrothermal pretreatment. Hydrothermal Processing in Biorefineries: Production of Bioethanol and High Added-Value Compounds of Second and Third Generation Biomass.

[12] Ruiz H.A., Rodríguez-Jasso M.A., Fernandes B.D., Vicente A.A., Teixeira J.A. (2013). Hydrothermal processing as an alternative for upgrading agriculture residues and marine biomass according to the biorefinery concept: A review. Renew. Sustain. Energy Rev..

[13] González-Muñoz M.J., Rivas S., Santos V., Parajó J.C. (2013). Aqueous processing of *Pinus pinaster* wood: Kinetics of polysaccharide breakdown. Chem. Eng. J..

[14] Rivas S., González-Muñoz M.J., Santos V., Parajó J.C. (2013). Production of furans from hemicellulosic saccharides in biphasic reaction systems. Holzforschung.

[15] Vila C., Romero J., Francisco J.L., Garrote G., Parajó J.C. (2011). Extracting value from *Eucalyptus* wood before kraft pulping: Effects of hemicelluloses solubilization on pulp properties. Bioresour. Technol..

[16] Vila C., Romero J., Francisco J.L., Santos V., Parajó J.C. (2012). On the recovery of hemicellulose before kraft pulping. BioResources.

[17] Werpy T., Petersen G. (2004). Top Value Added Chemicals from Biomass: Volume I—Results of Screening for Potential Candidates from Sugars and Synthesis Gas.

[18] Bozell J.J., Petersen G.R. (2010). Technology development for the production of biobased products from biorefinery carbohydrates—the US Department of Energy’s “Top 10” revisited. Green Chem..

[19] Peleteiro S., Rivas S., Alonso J.L., Santos V., Parajó J.C. (2016). Furfural production using ionic liquids: A review. Bioresour. Technol..

[20] Zeitsch K.J. (2000). The chemistry and technology of furfural and its many byproducts. Sugar Series.

[21] Zhang L., Xi G., Yu K., Yu H., Wang X. (2017). Furfural Production from biomass-derived carbohydrates and lignocellulosic residues via heterogeneous acid catalysts. Ind. Crop Prod..

[22] Peleteiro S., Santos V., Garrote G., Parajó J.C. (2016). Furfural production from *Eucalyptus* wood using an acidic ionic liquid. Carbohydr. Polym..

[23] Hoydonckx H.E., Van Rhijn W.M., Van Rhijn W., De Vos D.E., Jacobs P.A. (2007). Furfural and derivatives. Ullmann’s Encyclopedia of Industrial Chemistry.

[24] Peleteiro S., Raspolli-Galetti A., Antoneti C., Santos V., Parajó J.C. (2018). Manufacture of furfural from xylan-containing biomass by acidic processing of hemicellulose-derived saccharides in biphasic media using microwave heating. J. Wood Chem. Technol..

[25] Kim E.S., Liu S., Abu-Omar M.M., Mosier N.S. (2012). Selective conversion of biomass hemicellulose to furfural using maleic acid with microwave eating. Energy Fuels.

[26] Earle M.J., Seddon K.R. (2002). Ionic liquids: Green solvents for the future. ACS Symp. Ser..

[27] Huddleston J.G., Visser A.E., Reichert W.M., Willauer H.D., Broker G.A., Rogers R.D. (2001). Characterization and comparison of hydrophilic and hydrophobic room temperature ionic liquids incorporating the imidazolium cation. Green Chem..

[28] Stark A. (2011). Ionic liquids in the biorefinery: A critical assessment of their potential. Energy Environ. Sci..

[29] Guenic S.L., Delbecq F., Ceballos C., Len C. (2015). Microwave-assisted dehydration of D-xylose into furfural by diluted inexpensive inorganic salts solution in a biphasic system. J. Mol. Catal. A Chem..

[30] Yemis O., Mazza G. (2011). Acid-catalyzed conversion of xylose, xylan and xtraw into furfural. Bioresour. Technol..

[31] Prat D., Wells A., Hayler J., Sneddon H., McElroy C.R., Abou-Shehada S., Dunn P.J. (2015). CHEM21 selection guide of classical- and less classical-solvents. Green Chem..

[32] Tao F., Song H., Chou L. (2011). Efficient process for the conversion of xylose to furfural with acidic ionic liquid. Can. J. Chem..

[33] Peleteiro S., da Costa Lopes A.M., Garrote G., Parajó J.C., Bogel-Łukasik R. (2015). Simple and efficient furfural production from xylose in media containing 1-butyl- 3-methylimidazolium hydrogen sulfate. Ind. Eng. Chem. Res..

[34] Penín L., Santos V., del Río J.C., Parajó J.C. (2019). Assesment on the chemical fractionation of *Eucalyptus nitens* wood: Characterization of the products derived from the structural components. Bioresour. Technol..

[35] Overend R.P., Chornet E. (1987). Fractionation of lignocellulosics by steam-aqueous petreatments. Philos. Trans. R. Soc. A.

[36] Peleteiro S., Santos V., Parajó J.C. (2016). Furfural production in biphasic media using an acidic ionic liquid as a catalyst. Carbohydr. Polym..

[37] Wang S., Zhao Y., Lin H., Chen J., Zhu L., Luo Z. (2017). Conversion of C5 carbohydrates into furfural catalyzed by a Lewis acidic ionic liquid in renewable γ-valerolactone. Green Chem..

[38] Xia Q., Peng H., Zhang Y., Fu G., Liu Y., Xiao Z., Huang L., Bi H. (2021). Microwave-assisted furfural production from xylose and bamboo hemicellulose in a biphasic medium. Biomass Conv. Bioref..

[39] Matsagar B.M., Munshi M.K., Kelkar A.A., Dhepe P.L. (2015). Conversion of concentrated sugar solutions into 5-hydroxymethyl furfural and furfural using Brönsted acidic ionic liquids. Catal. Sci. Technol..

[40] Lin H., Chen J., Zhao Y., Wang S. (2017). Conversion of C5 Carbohydrates into furfural catalyzed by SO_3_H-functionalized ionic liquid in renewable γ-valerolactone. Energy Fuels.

[41] López M., Rivas S., Vila C., Santos V., Parajó J.C. (2020). Performance of 1-(3-sulfopropyl)-3-methylimidazolium hydrogen sulfate as a catalyst for hardwood upgrading into bio-based platform chemicals. Catalysts.

